# Comparing Work Experiences of Internal Medicine Physicians in Veterans Affairs and Non-Federal Hospitals: A National Survey

**DOI:** 10.1007/s11606-025-09797-9

**Published:** 2025-09-04

**Authors:** Sarah K. Gualano, M. Todd Greene, Nathan Houchens, Richard J. Schildhouse, David Ratz, Karen E. Fowler, Sanjay Saint

**Affiliations:** 1 Medicine Service, Veterans Affairs Ann Arbor Healthcare System, Ann Arbor, MI USA; 2 Department of Internal Medicine, University of Michigan Medical School, Ann Arbor, MI USA; 3 Center for Clinical Management Research, Veterans Affairs Ann Arbor Healthcare System, Ann Arbor, MI USA; 4 Division of Cardiovascular Medicine, University of Michigan, Cardiovascular Center 2 A 192G, 1500 E. Medical Center Dr., SPC 5869, Ann Arbor, MI 48109-5869 USA

**Keywords:** burnout, well-being, work experience, workload, work environment

## Abstract

**Background:**

Fewer physicians are pursuing careers in internal medicine due to workload, work-life integration, and documentation pressures. Integrated health systems like the Department of Veterans Affairs (VA) offer an alternative work model that may affect physicians’ experiences differently compared to the private sector.

**Objective:**

To examine and compare the self-reported workplace experiences among VA and non-VA internists.

**Design:**

A cross-sectional survey.

**Participants:**

A random sample of 1421 internal medicine physicians across the USA, with oversampling of hospitalists and VA physicians. Surveys were completed between June 2023 and May 2024. Of the total of 629 (44.3%) physicians responding, 618 indicated working in a VA or non-VA hospital.

**Main Measures:**

Our primary outcomes were several professional environment factors. Multivariable logistic regression was used to assess relationships between professional environment factors and practicing at the VA (vs. non-VA). All models were adjusted for sex, race, and years practicing as an internist.

**Key Points:**

The odds of time engaging with health insurance companies (OR = 0.26; *P* < 0.001), medical record issues (OR = 0.44; *P* < 0.001), and malpractice concerns (OR = 0.56; *P* < 0.001) were significantly lower among VA internists compared with non-VA internists. VA internists were significantly less likely to report issues concerning work-life balance (OR = 0.63; *P* = 0.01).

**Conclusions:**

VA internists had reduced odds of some non-clinical administrative tasks and greater odds of better work-life balance. The VA’s minimal engagement with insurance tasks and litigation concerns appears to present a compelling work environment.

**Supplementary Information:**

The online version contains supplementary material available at 10.1007/s11606-025-09797-9.

## INTRODUCTION

An engaged and fulfilled healthcare workforce is a crucial requirement for effective care delivery.^1–3^ Physician satisfaction and engagement have emerged as vital performance metrics for healthcare institutions and are indicators of workforce stability.^4–10^ Additionally, job-related factors (e.g., type of work, workload, and burnout), autonomy, quality of management, supervisory support, and leadership have been shown to influence physician satisfaction and turnover in both VA and non-VA physicians.^11–14^

General internists are the backbone of care delivery, serving as the first point of contact for patients and staff as well as coordinators of complex specialty care in the inpatient and outpatient settings. Current trends of practicing physicians to reduce hours or leave practice will contribute to physician shortages.^15^ Meanwhile, career choices of internal medicine residents are concerning, as fewer trainees are opting for careers in general internal medicine compared to medical subspecialties. A 2021 study of internal medicine post-graduate year 3 residents demonstrated only 25% planned on practicing as internists (general internal medicine 9.4%, hospital medicine 15.1%), half the number in a similar survey a decade earlier.^16,17^ This is notable given the Association of American Medical Colleges projected shortage of nearly 50,000 primary care physicians by 2032.^16,18^ Workload, work-life integration, and documentation pressures have been described as reasons for choosing other careers.^19^ A deeper understanding of the work experiences of internists across various healthcare delivery models may provide insights into practice environments which may appeal to a broader group of physicians.

Integrated health systems such as the Veterans Health Administration (VHA), the healthcare branch of the Department of Veterans Affairs (VA), offer physicians an alternative work model and environment compared to the predominately fee-for-service private sector or academic practices. The VA is the largest integrated healthcare system in the USA, employing more than 300,000 healthcare professionals at more than 170 VA medical centers and 1063 outpatient clinical sites.^20,21^ This includes more than 26,000 physicians working at the VA as direct employees, trainees, fee-basis providers, or under contract.^22^ Past studies exploring the work environment differences between VA and the private sector were conducted prior to substantial changes in care delivery within the VA. In 2010, the VA launched the patient-centered medical home strategy or patient-aligned care teams (PACT) model for primary care delivery.^23^ The PACT “teamlets” of four include a primary care physician, nurse care manager, clinical associate or licensed practical nurse, and medical support assistant, with a maximum panel size of 1200 patients per teamlet.^24^ The passage of the Maintaining Internal Systems and Strengthening Integrated Outside Networks (MISSION) Act in 2018 allowed Veterans greater choice in care site, either within the community or the VA. The act also resulted in notably greater administrative tasks for PACT physicians, as they remained responsible for coordination of care for Veterans receiving care from community providers.^25^ It remains unclear whether the development of PACT or the passage of the MISSION Act has had a positive or negative impact on providers within the VA. We thus sought to examine self-reported workplace environment, experiences, and perspectives between VA-based and non-VA internists through a contemporary national survey. Our goal was to compare the extent to which various professional environment factors (e.g., workload, administrative demands, malpractice issues, leadership support, patient relationships, and work-life balance)  differed between physicians working in VA and non-VA settings.

## METHODS

### Study Design and Data Collection

We conducted a nationwide cross-sectional survey of internists practicing in the USA to describe the workplace environment, community, and personal factors including levels of burnout. Our survey was first piloted with a sample of 19 internal medicine physicians from various facilities. The survey was then revised for use in this study, and a paper survey was sent to a randomly selected sample of practicing internists identified through Physician Professional Data, a national physician database maintained by the American Medical Association. Between June 2023 and May 2024, 1611 surveys were mailed, oversampling hospitalists (30%) and VA internists (30%). The remaining 40% were internists in equal proportions within the four geographic regions of the USA: Northeast, Midwest, South, and West. A final sample of 1421 internists was achieved after removing surveys that were returned as undelivered or those in which the respondent indicated they had retired.

Validated methods were used to distribute the survey: (1) a pre-notification email (when email address available) or letter; (2) an initial mailing information letter with a copy of the survey, a postage-paid return envelope, and a $20 incentive; (3) a reminder email or letter to non-respondents after approximately 2 weeks; and (4) additional survey mailings to non-respondents after 1 month, 2 months, and 3 months.^26^ A link to an online version of the survey was included for participants who preferred to complete the survey electronically.

Completed surveys were anonymous and did not include personally identifiable information. The study was reviewed and deemed exempt from regulation by the University of Michigan Institutional Review Board (HUM00228326).

### Survey Measures

The survey (Supplement 1) included 42 questions (many with sub-questions) designed to assess the workplace environment, physician well-being, and professional burnout.^27^ The MBI-HSS was scored according to the standard published scoring algorithm. Of note, there is a lack of consensus on the operational definition of burnout.^28,29^ We used two dichotomous outcomes: (1) “extreme burnout” was defined as scores meeting set thresholds in all three burnout domains (i.e., emotional exhaustion ≥ 27, depersonalization ≥ 10, and personal accomplishment ≤ 33), and (2) “at least one manifestation of burnout” (i.e., emotional exhaustion ≥ 27 or depersonalization ≥ 10).^29^ Our primary outcomes included 13 professional environment factors hypothesized to contribute to burnout: malpractice concerns, time working with insurance companies, interface with the electronic health record (EHR), workload levels, professional autonomy, staffing and services support, feelings of working at the top of license, discrimination and perceptions of value from patients and leadership, and life and financial stressors. All 13 factors were assessed via the prompt, “To what extent do you believe the following items contribute to physician burnout?” and were based on a 5-point Likert scale ranging from “Not at all” (1) to “A great deal” (5). Three additional primary outcomes were also assessed: two regarding malpractice (“have you ever been sued for malpractice (yes/no)?” and “has a friend or colleague been sued for medical malpractice (yes/no)?”) and one regarding work-life balance (“On a scale from 1–10 (1 = not at all, 10 = all the time), how much does work interfere with you participating in the social activities and hobbies you'd like to do?”). Demographic data including sex, race/ethnicity, marital and family status, time in practice, and type, setting, and context of clinical care were also collected.

### Statistical Analysis

Descriptive statistics were calculated for physician characteristics. Respondents were not required to answer all questions, and results for each question may have come from a different number of respondents. Missing responses were generally low and not imputed. To investigate associations between practicing at the VA (vs. non-VA settings) and various professional environment, community, and personal factors, multivariable logistic regression was used. The primary predictor variable was practicing at the VA (coded 1) vs. other practice settings (coded 0). Professional environment factors hypothesized to contribute to burnout were assessed on a 5-point Likert scale and were treated as ordinal outcome variables. All regression models were adjusted for the number of years practicing as an internist, working as a hospitalist, sex, and race. A *P*-value < 0.05 was considered statistically significant. SAS version 9.4 (Cary, NC) was used for all analyses.

## RESULTS

Of the 1421 physicians invited to participate, 629 (44.3%) completed the survey. A majority (74.9%) of surveys were completed on paper, with the remainder completed electronically. Our final analytic sample included 618 physicians that indicated their work affiliation. A total of 186 (30.1%) worked for the VA. Among the 432 (69.9%) working at a non-VA medical center or clinic, 277 (64.1%) worked for an academic medical center or clinic, 86 (19.9%) for a community medical center or clinic, and 69 (16.0%) other types of facilities. Physician characteristics are provided in Table 1. Among all respondents, 59.8% were male, 57.6% were white, and 84.9% were married or living as if married. The distributions of sex (*P* = 0.29), race (*P* = 0.28), and marital status (*P* = 0.65) did not differ between VA and non-VA physicians. A greater proportion of VA physicians provided primary care (VA = 70.4% vs. non-VA 40.7%, *P* < 0.001), whereas a greater proportion of non-VA physicians considered themselves hospitalists (VA = 21.0% vs. non-VA 60.4%, *P* < 0.001). VA physicians reported practicing as an internist for longer (VA = 26.4 years vs. non-VA = 20.6 years, *P* < 0.001). Conversely, non-VA physicians reported working more hours per week (VA = 47.6 h vs. non-VA = 58.6 h, *P* < 0.001). A total of 9.8% of respondents met thresholds for “extreme burnout” and 61.5% for “at least one manifestation of burnout.” There was no difference in burnout prevalence among VA and non-VA internists for either burnout definition (“extreme burnout” — VA (10.9%) vs. non-VA (9.3%), *P* = 0.56; “one manifestation of burnout” — VA (58.7%) vs. non-VA (62.7%), *P* = 0.35).

**Table 1:** Characteristics of Internal Medicine Physician Study Participants

Characteristic	VA (*n* = 186)	Non-VA (*n* = 432)	*P*-value
	**No. (%)**	**No. (%)**	
**Burnout**			
Extreme burnout	20/184 (10.9)	40/429 (9.3%)	0.55
One manifestation burnout	108/184 (58.7)	269/429 (62.7%)	0.35
**Sex**			
Male	106/185 (57.3)	270/430 (62.8)	0.29
Female	79/185 (42.7)	156/430 (36.3)	
Other/prefer not to answer	0 (0)	4/430 (0.09)	
**Race**			
White	102/182 (56.0)	260/427 (60.9)	0.28
Asian	61/182 (33.5)	127/427 (29.7)	
Black or African American	10/182 (5.5)	21/427 (4.9)	
Native Hawaiian or Pacific Islander	2/182 (1.1)	0/427 (0)	
American Indian or Alaskan Native	0/182 (0)	1/427 (0.2)	
Other	7/182 (3.9)	18/427 (4.2)	
**Marital status**			
Married or living as if married	163/186 (87.6)	370/430 (86.1)	0.65
Single, never married	7/186 (3.8)	26/430 (6.1)	
Divorced	11/186 (5.9)	22/430 (5.1)	
Separated	2/186 (1.1)	8/430 (1.9)	
Widowed	3/186 (1.6)	4/430 (0.9)	
**Children under age 18 years**			
Yes	49/185 (26.5)	216/431 (50.1)	< 0.001
**Clinical work setting**			
Inpatient setting only	11/114 (9.7)	63/275 (22.9)	0.01
Outpatient setting only	36/114 (31.6)	57/275 (20.7)	
Both inpatient and outpatient	3/114 (2.6)	11/275 (4.0)	
Other	64/114 (56.1)	144/275 (52.4)	
Role*			
Primary care physician	131/186 (70.4)	255/430 (40.7)	< 0.001
Hospitalist	39/186 (21.0)	259/429 (60.4)	< 0.001
**Time spent**			
Years in practice, mean (SD)	26.4 (8.2)	20.6 (9.6)	< 0.001
Hours working per week, mean (SD)	47.6 (14.7)	58.6 (21.4)	< 0.001

^*^These percentages do not equal 100%, because two separate questions with dichotomous responses were posed: (1) Do you consider yourself a hospitalist? (2) Do you provide primary care?

Multivariable regression analysis demonstrated professional environment factors which differed between VA and non-VA respondents (Table 2). The odds of several professional environment factors contributing to physician burnout were significantly reduced among VA physicians including: time devoted to engaging with health insurance companies (OR = 0.26, 95%CI 0.18 to 0.37, *P* < 0.001), issues working with the EHR (OR = 0.44, 95%CI 0.31 to 0.62, *P* < 0.001), personal life stressors (e.g., childcare) (OR = 0.66, 95%CI 0.47 to 0.93, *P* = 0.02), and financial stressors (OR = 0.67, 95%CI 0.48 to 0.96, *P* = 0.03). VA internists had significantly lower odds of concerns related to being sued for malpractice (OR = 0.56, 95%CI 0.40 to 0.80, *P* < 0.001) and having a friend or close colleague sued for medical malpractice (OR = 0.49, 95%CI 0.33 to 0.74, *P* < 0.001), but there was no significant difference in odds of having been personally sued for medical malpractice. VA internists were significantly less likely to report work interfering with participation in social activities and personal hobbies (0.63, 95%CI 0.45 to 0.88, *P* = 0.01).

**Table 2:** Multivariable Regression Associations Between Practicing at the VA (vs. non-VA) and Professional Environment, Community, and Personal Factors

**Question**	OR	95% CI		*P*
**Professional environment factors**				
Concerns related to being sued for medical malpractice ^a^	0.56	0.40	0.80	< 0.001
Time devoted to engaging with health insurance companies ^a^	0.26	0.18	0.37	< 0.001
Issues working with the electronic health record ^a^	0.44	0.31	0.62	< 0.001
High workload (e.g., census is too high) ^a^	0.87	0.61	1.24	0.44
Not having enough autonomy over workload ^a^	1.03	0.73	1.46	0.86
Not having the needed support (e.g., support staff and/or services) ^a^	1.24	0.88	1.76	0.22
Feeling like you are not working at the top of your license ^a^	1.17	0.83	1.65	0.36
Feeling undervalued by senior leadership in my organization ^a^	1.27	0.90	1.80	0.17
Feeling undervalued by patients ^a^	0.79	0.56	1.12	0.18
Personal life stressors (e.g., childcare) ^a^	0.66	0.47	0.93	0.02
Financial stressors ^a^	0.67	0.48	0.96	0.03
Feelings of being discriminated against by employer ^a^	1.05	0.73	1.52	0.78
Feelings of being discriminated against by patients ^a^	0.97	0.67	1.40	0.88
Friend or a close colleague has been sued for medical malpractice (y/n)	0.49	0.33	0.74	< 0.001
Have been sued for medical malpractice (y/n)	0.80	0.53	1.20	0.29
On a scale from 1–10 (1 = not at all, 10 = all the time), how much does work interfere with you participating in the social activities and hobbies you'd like to do?	0.63	0.45	0.88	0.01

All models adjusted for race, sex, working as a hospitalist, and number of years practicing as an internist. Odds ratios reflect associations related to practicing at the VA (coded 1) vs. non-VA (coded 0). As such, odds ratios reflect the odds of being in a higher category within each scale given practicing at the VA

*OR*, odds ratio; *CI*, confidence interval

^a^Responses based on 5- Likert scales (ranging from 1 (not at all) to 5 (a great deal)) treated ordinally

## DISCUSSION

The VA is recognized for its leadership in quality, safety, and performance improvement, along with specialized clinical services tailored to Veteran patients (e.g., mental health, primary care).^30–33^ In this national cross-sectional study involving a randomly selected sample of general internists across the USA, we identified several statistically significant differences in the work environment of physicians whose primary practice is within the VA compared to those practicing outside the VA. There were several notable differences in reported systems and environment of practice between VA and non-VA physicians. The discrepancy in time engaging with insurance companies between the two groups was both significant and predictable, given the capitated care model of the VA versus fee-for-service healthcare systems. As frustrations with insurance companies rise, the appeal of a practice that minimizes time spent on insurance communication may grow.^34^

The EHR is a well-described contributor to workplace frustration and burnout, and EHR-related distress has been identified as a driver of decreasing interest in primary care as a career.^35^ At the time of this study, the vast majority of VA systems remained on the VA’s Computerized Patient Record System (CPRS), which, despite its age and technical limitations, is highly rated for ease of use, vendor support, connectivity, and clinical usefulness compared to private EHR vendors.^36^ Our findings that VA internists noted fewer issues when working in CPRS compared to non-VA internists who may use a variety of commercially available programs align with these previous surveys. As the VA continues to implement a commercially available EHR product across the enterprise, it is noted that initial difficulties substantial enough to require a deployment “reset” were necessary to implement critical product improvements, which delayed deployments for 23 months.^37–39^ Whether the ongoing transition to a commercially available EHR fully addresses concerns from early adopter sites remains to be seen.^40–43^ If not, there is a distinct possibility that the difference in experience between VA and non-VA may narrow or even reverse.

VA internists were less likely to experience interference with social activities and life outside of work compared to non-VA internists. The ability to enjoy a life outside of work is a predictor of overall career satisfaction.^19,44^ Veterans Affairs careers are often described as having greater work-life integration, likely due to smaller patient panels and the relative lack of non-clinical administrative tasks present in other practice models.^21^ Professional resilience is bolstered by access to leisure activities and a clear demarcation between work and personal life.^45^

The VA represents a unique professional environment with minimal exposure to much of the regulatory and financial requirements and pressures in other practice settings.^46,47^ In our study, VA internists reported fewer personal life and financial stressors compared to non-VA internists. This finding may be surprising given the perceived lower financial benefits within the federal system; however, financial compensation and incentives are not the sole driver of workplace satisfaction.^48^ The VA operates as a predominant capitated system, with budgets determined in part by patient- and facility-level complexity.^49^ Decreasing physician reimbursement and changing payment models may be adding stress to physicians working in a traditional fee-for-service model.^50^

The impact of malpractice, whether personally experienced or observed through a colleague, was significantly less pronounced for those practicing within the VA system. Interestingly, this finding was in spite of a similar reported rate of malpractice. Physicians working at the VA are covered under the Federal Tort Claims Act, rather than an individual or institutional group practice plan.^51^ The implication of this type of coverage is they may not be sued as an individual, with the associated negative impact, but rather lawsuits are handled by the Office of General Council. Malpractice can lead to emotional trauma among healthcare providers and have a lasting negative impact on both professional and personal lives. The term “second victim” has been used to describe the healthcare employee involved in malpractice claims and adverse patient outcomes.^19–21^ Identifying the specific reasons for the seeming conflict between perception of impact and reported rate of malpractice is beyond the scope of the current study.

There were no statistically significant differences between VA and non-VA internists for several workload metrics, including having a large census, lack of autonomy over workload, not having adequate support staff or services, and not working at the top of your license. Quantitatively, those working at the VA reported working nearly 10 fewer hours per week than non-VA internists. The variation in implementation and staffing of PACT, particularly specialty PACT such as Geriatric PACT and Homeless PACT, may be a contributing factor to the lack of difference seen between the two groups. The overall impact of a PACT on provider effectiveness and well-being remains mixed.^52–67^

In the present study, we did not detect a statistically significant difference in burnout between VA and non-VA internists, despite work environment differences which were seen. Studies completed prior to the passage of the MISSION Act suggest physician burnout in the VA may be lower than reported in non-VA settings. One small study—specifically focused on general internal medicine—demonstrated a signal for less burnout in VA vs non-VA internists (17% vs. 40%); however, the limited number of respondents (VA = 45, non-VA = 534) may reduce the generalizability of this analysis.^19^ One pre-MISSION Act and pre-COVID-19 study of the rate of burnout specifically among all PACT members did note a trend towards lower reported burnout over time, though whether this trend continues is unknown.^68^ While the outcomes we examined were hypothesized to contribute to burnout, there are likely other organizational and operational factors which were not included in the survey, thus blunting our ability to detect a difference between the two groups.

These findings should be considered in the context of several limitations. While the factors represented in the survey captured established aspects of a work environment, there may be factors which remain unrecognized and thus not represented or examined. The use of a survey instrument may not provide a nuanced representation of physician work life that a qualitative study may achieve. Although we achieved a high response rate for a national survey of clinician attitudes (with over-sampling of VA physicians), our respondents may not represent the perspective of physicians who did not participate. The respondents’ average time in practice was relatively high at 23 years, which could skew responses towards those of mid- and late-career physicians. The survey did not directly ask for details regarding compensation (salary vs. fully fee-for-service or a hybrid), specific workload targets, or patient expectations and reviews. Lastly, as a cross-sectional study, our results may be susceptible to reverse causality and thus limit causal inferences.

Limitations notwithstanding, the reduced impact of malpractice, a more favorable EHR, and greater odds of a favorable work-life balance were the primary work experience differences identified between VA and non-federal employees in this national survey. Reduced time spent on non-clinical administrative tasks may allow for more time available for meaningful physician–patient relationships. Despite variable success in fully implementing the PACT model, successful systems and clinics have demonstrated that working together with a core team of collaborating health professionals may be a path forward for other non-federal systems.^62^ These benefits and positive work experiences could be more broadly shared not only to make a career in internal medicine more appealing, but also to enhance recruitment of physicians to the VA.^69^

## Supplementary Information

Below is the link to the electronic supplementary material.

Supplementary file1 (PDF 343 KB)

## Data Availability

Deidentified data will be made available upon reasonable request and ethical approval (mtgreene@med.umich.edu, MTG).

## Declarations:

### Disclaimer:

The funder had no role in the design, execution, analyses, interpretation of data, or decision to publish results.
