# Supplementary material for: Comparing Work Experiences of Internal Medicine Physicians in Veterans Affairs and Non-Federal Hospitals: A National Survey
Source: J Gen Intern Med. 2025 Sep 4;41(2):437–44. doi: 10.1007/s11606-025-09797-9 (PMC12894548; doi:10.1007/s11606-025-09797-9)
Supplement: Supplementary file 1 — Supplementary file1 (PDF 343 KB) [file 11606_2025_9797_MOESM1_ESM.pdf]

THE INTERNIST WELLNESS SURVEY

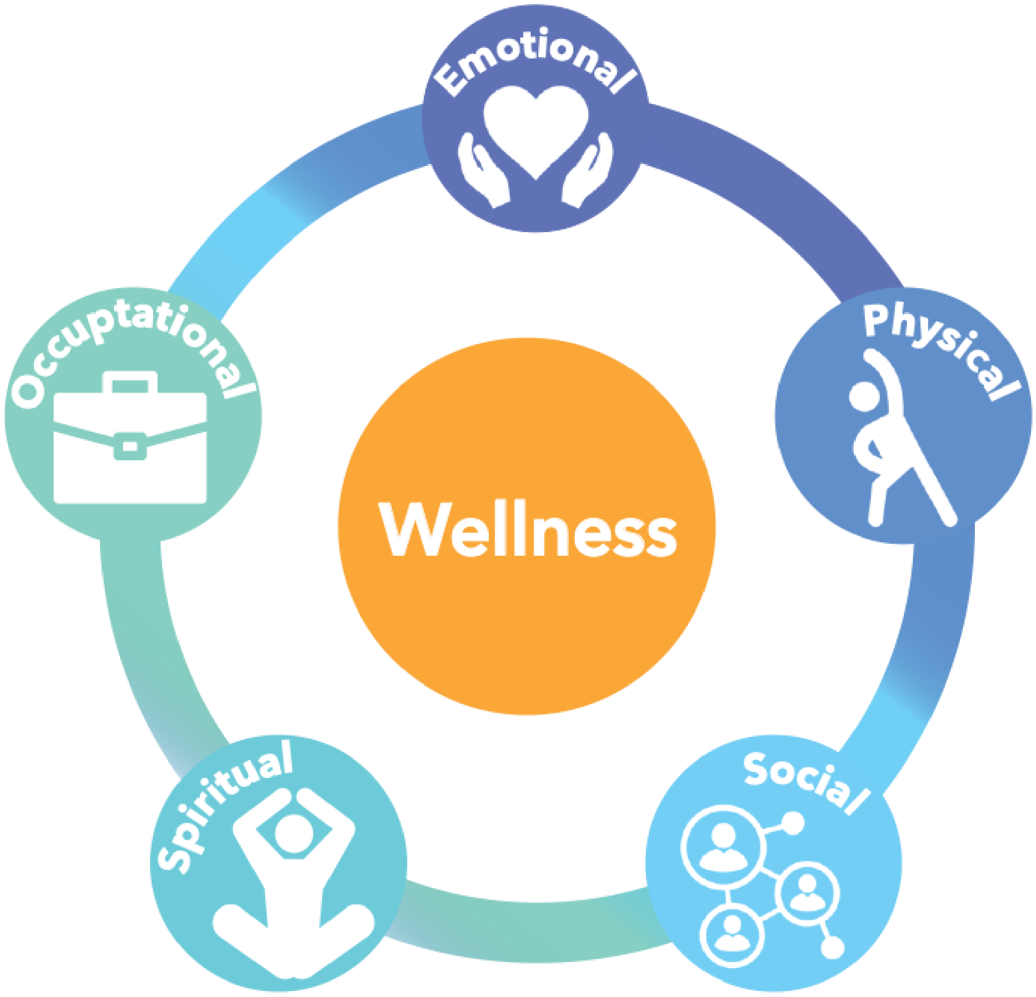

The purpose of this survey is to assess the level of wellness of internists across the United States. This study is being conducted by researchers from the University of Michigan.

Your participation in this survey is **completely voluntary** and should take **approximately 15 minutes**. By returning this survey, you are agreeing to participate in this research study. All responses will be kept anonymous and confidential. No identifying information will be collected. Your opinion is very valuable in helping us describe internist wellness patterns. Please use a blue or black pen to complete this survey.

SECTION A - MINDFULNESS

1. Below is a collection of statements about your everyday experience. Using the 1-6 scale below, please indicate how frequently or infrequently you currently have each experience. Please answer according to what really reflects your experience rather than what you think your experience should be. Please treat each item separately from every other item.

	Almost Never	Very Infrequently	Somewhat Infrequently	Somewhat Frequently	Very Frequently	Almost Always
A. It seems I am "running on automatic," without much awareness of what I'm doing.	1	2	3	4	5	6
B. I rush through activities without being really attentive to them.	1	2	3	4	5	6
C. I find myself preoccupied with the future or the past.	1	2	3	4	5	6
D. I find myself doing things without paying attention.	1	2	3	4	5	6

2. How often do you practice <u>any form</u> of meditation (for example, breathing exercises, visualization, yoga)?
   - ☐1 Every day
   - ☐2 A few times per week
   - ☐3 Once per week
   - ☐4 A few times per month
   - ☐5 Once per month
   - ☐6 A few times per year
   - ☐7 Never

3. How many years have you engaged in meditation?
   - ☐1 Less than 1 year
   - ☐2 1-5 years
   - ☐3 6-10 years
   - ☐4 >10 years
   - ☐5 N/A – I do not practice meditation

SECTION B – RELIGIOUS/SPIRITUAL PRACTICES

The next few questions are about your religious/spiritual practices.

4. How often do you attend religious services?
   - ☐1 Never
   - ☐2 Less than once a year
   - ☐3 Several times a year
   - ☐4 About once per month
   - ☐5 2 to 3 times per month
   - ☐6 Nearly every week
   - ☐7 Several times per week

5. What is your religious affiliation?

- ☐1 Buddhist
- ☐2 Hindu
- ☐3 Jewish
- ☐4 Muslim
- ☐5 Roman Catholic
- ☐6 Eastern Orthodox
- ☐7 Protestant
- ☐8 Other Christian
- ☐9 Other, please specify: ___________________________________
- ☐10 None

6. How often do you pray privately in places other than at church, synagogue or other place of worship?

- ☐1 Never
- ☐2 1 to 2 times per month
- ☐3 1 to 2 times per week
- ☐4 Approximately once per day
- ☐5 More than once per day

7. Do you believe in God or another higher power?

- ☐1 Yes
- ☐2 No
- ☐3 Undecided
- ☐4 Prefer not to answer

(No, Undecided, Prefer not to answer:) **Skip to question 8**

a. If yes, do you believe that God or another higher power is in control of the universe?

- ☐1 Yes
- ☐2 No
- ☐3 Undecided
- ☐4 Prefer not to answer

8. Do you believe there is a life after death?

- ☐1 Yes
- ☐2 No
- ☐3 Undecided
- ☐4 Prefer not to answer

9. To what extent do you consider yourself a spiritual person?

- ☐1 Not spiritual at all
- ☐2 Slightly spiritual
- ☐3 Moderately spiritual
- ☐4 Very spiritual
- ☐5 Prefer not to answer

10. Please indicate your level of agreement with the following statements:

	**Strongly agree**	**Somewhat agree**	**Neither agree nor disagree**	**Somewhat disagree**	**Strongly disagree**
A. "I try hard to carry my religious beliefs over into all my other dealings in life."	1	2	3	4	5
B. "I have a strong sense of purpose in my **life**."	1	2	3	4	5
C. "I have a strong sense of purpose in my **work**."	1	2	3	4	5

11. "Sacred Moments" are described as deeply meaningful, memorable, and sometimes spiritual moments shared between physicians and patients – as if time stood still. These may happen spontaneously during times of crisis or sadness, or conversely during times of great joy.

a. Have you ever experienced a Sacred Moment with a patient?

☐1 Yes
☐2 No → **Skip to question 12**

b. <u>If yes</u>, how often do you experience Sacred Moments with a patient?

☐1 Weekly
☐2 Monthly
☐3 A few times per year
☐4 Yearly
☐5 A few times in my career

c. How often do you talk with your colleagues about your Sacred Moment experiences?

☐1 Never
☐2 Rarely
☐3 Sometimes
☐4 Often
☐5 Always

d. Please indicate how much you agree or disagree with the following statement. "Experiencing a Sacred Moment with a patient helps me feel less burned out."

☐1 Strongly agree
☐2 Somewhat agree
☐3 Neither agree nor disagree
☐4 Somewhat disagree
☐5 Strongly disagree

SECTION C – PHYSICAL AND SOCIAL ACTIVITIES

The next section will ask questions about your participation in physical and social activities.

12. During the **last 7 days**, on how many days did you participate in any exercise or other physical activities?

    ________ **days** ☐ No exercise or physical activities in the last 7 days → **Skip to question 14**

13. How much time did you usually spend doing exercise or other physical activities on one of those days?

    ________ **hours** _________ **minutes per day** ☐ Don't know/Not sure

14. Within the **past 30 days**, indicate the number of times you've done any of the following.

	**Never**	**1 time**	**2 to 3 times**	**4 to 5 times**	**More than 5 times**
A. Attended or participated in an in-person social event.	1	2	3	4	5
B. Attended or participated in a virtual social event.	1	2	3	4	5
C. Participated in an individual-based, relaxing event (e.g., read for pleasure, day spa, yoga, played a musical instrument).	1	2	3	4	5
D. Participated in any other personal hobby.	1	2	3	4	5
E. Did volunteer work.	1	2	3	4	5

15. On a scale from 1-10, how much does work interfere with you participating in the social activities and hobbies you'd like to do?

1	2	3	4	5	6	7	8	9	10
Not at all									All the time

SECTION D –WELL-BEING

16. Please indicate how much you agree or disagree with the following statements.

	**Strongly agree**	**Agree**	**Neither agree nor disagree**	**Disagree**	**Strongly disagree**
A. My work schedule leaves me enough time for my personal/family life.	1	2	3	4	5
B. I feel as if I can someday master my work.	1	2	3	4	5
C. I have great autonomy over my work.	1	2	3	4	5
D. I am paid less than I should be making.	1	2	3	4	5

17. Below are 22 statements of job-related feelings. Please read each statement carefully and decide if you ever feel this way about your job. If you have *never* had this feeling, circle the number "0" (zero). If you have had this feeling, indicate *how often* you feel it by circling a number (from 1 to 6) that best describes how frequently you feel that way.

Q17 Questions Redacted Due to a Survey Licensing Agreement.

The 22 items came from the Maslach Burnout Inventory – Human Services Survey for Medical Personnel Copyright ©1981, 2016 Christina Maslach & Susan E. Jackson. All rights reserved in all media. Published by Mind Garden, Inc., www.mindgarden.com.

18. Overall, based on your definition of burnout, how would you rate your level of burnout?

- ☐1 I enjoy my work. I have no symptoms of burnout.
- ☐2 Occasionally I am under stress, and I don't always have as much energy as I once did, but I don't feel burned out.
- ☐3 I am definitely burning out and have one or more symptoms of burnout, such as physical and emotional exhaustion.
- ☐4 The symptoms of burnout that I'm experiencing won't go away. I think about frustration at work a lot.
- ☐5 I feel completely burned out and often wonder if I can go on. I am at the point where I may need to seek some sort of help.

If you feel like you are experiencing feelings of burnout, there are resources available to help you. For example, the Physician Support Line is a free, confidential & anonymous service available Monday – Friday (except federal holidays) from 8:00 AM to 12:00 AM. **1 (888) 409-0141; https://www.physiciansupportline.com/**.

19. To what extent do you believe the following items contribute to **physician burnout**.

	Not at all	A little bit	Somewhat	Quite a bit	A great deal
A. Concerns related to being sued for medical malpractice.	1	2	3	4	5
B. Time devoted to engaging with health insurance companies.	1	2	3	4	5
C. Issues working with the electronic health record.	1	2	3	4	5
D. High workload (e.g., census is too high).	1	2	3	4	5
E. Not having enough autonomy over workload.	1	2	3	4	5
F. Not having the needed support (e.g., support staff and/or services).	1	2	3	4	5
G. Feeling like you are not working at the top of your license.	1	2	3	4	5
H. Feeling undervalued by senior leadership in my organization.	1	2	3	4	5
I. Feeling undervalued by patients.	1	2	3	4	5
J. Personal life stressors (e.g., childcare).	1	2	3	4	5
K. Financial stressors.	1	2	3	4	5
L. Feelings of being discriminated against by employer.	1	2	3	4	5
M. Feelings of being discriminated against by patients.	1	2	3	4	5

SECTION E - SUPPORT

20. To what extent do you agree or disagree with each of the following statements about your immediate PHYSICIAN supervisor? If you have more than one, please think about whomever supervises your clinical time.

	Strongly disagree	Disagree	Neither agree nor disagree	Agree	Strongly agree	Do not know/ Not applicable
A. My immediate physician supervisor empowers me to do my job.	1	2	3	4	5	6
B. My immediate physician supervisor treats me with respect and dignity.	1	2	3	4	5	6

21. How would you rank the level of support you receive from the following?

	Poor	Fair	Good	Very good	Excellent
A. Your hospital's senior leadership.	1	2	3	4	5
B. Your coworkers.	1	2	3	4	5
C. Your family.	1	2	3	4	5
D. Your friends.	1	2	3	4	5

22. Do you have any professional mentors?
    - ☐1 Yes
    - ☐2 No → **Skip to question 23**

    a. If yes, how would you rank the level of support you receive from your mentor(s)?
    - ☐1 Poor
    - ☐2 Fair
    - ☐3 Good
    - ☐4 Very good
    - ☐5 Excellent

23. Do you believe that "human factors" – defined "as the scientific discipline concerned with the understanding of interactions among humans and other elements of a system in order to optimize human well-being and overall system performance" – has a role to play in enhancing your wellbeing as an internist?
    - ☐1 Yes
    - ☐2 No
    - ☐3 Not sure

    If yes, do you have any examples or suggestions for how?

    ____

    ____

    ____

SECTION F – WORK EXPERIENCE

24. How long have you been practicing as an internist?

    _____________ **Years** _____________ **Months**

25. Please indicate below in which settings you perform clinical work. (Select only one)
    - ☐1 Outpatient setting only
    - ☐2 Inpatient setting only
    - ☐3 Both the inpatient and outpatient setting
    - ☐4 Other setting (please specify) ______________________________________

26. If you work in both the inpatient and outpatient setting, in which **setting** do you spend the majority of your clinical time? (Select only one)

- ☐1 Inpatient setting
- ☐2 Outpatient setting
- ☐3 Equal time in both settings
- ☐4 N/A - I do not work in both settings

27. In what **type of facility** do you spend the majority of your clinical time? (Select only one)

- ☐1 VA Medical Center or Clinic
- ☐2 Academic Medical Center or Clinic
- ☐3 Community Medical Center or Clinic
- ☐4 Other (please specify) ___________________

28. How many hours do you work in a typical week (for hospitalists, please answer for a week when you are on service)? ___________

29. Of your total work hours, approximately what % of your time is spent on the following:

_______ Outpatient Care

_______ Inpatient Care

_______ Administrative Duties

_______ Research

_______ Teaching

_______ Other, please specify: ________________________

30. Do you consider yourself a hospitalist?

- ☐1 Yes
- ☐2 No

31. Do you provide primary care?

- ☐1 Yes
- ☐2 No → **Skip to question 32**

a. If yes, what is your approximate patient panel size? _____________

32. In what state do you practice medicine? If more than one state, please indicate the one in which you practice the **most**. ____________________ State

33. In what country did you complete medical school? ____________________ Country

34. Has a friend or a close colleague of yours ever been sued for medical malpractice?

- ☐1 Yes
- ☐2 No
- ☐3 Don't Know

35. Have you ever been sued for medical malpractice?

- ☐1 Yes
- ☐2 No

36. Have malpractice concerns made you consider quitting medicine?

- ☐1 Yes
- ☐2 No

SECTION G - DEMOGRAPHICS

Lastly, we would like to know a little bit about you. Please answer the following questions.

37. Do you identify as transgender?

- ☐1 Yes
- ☐2 No
- ☐3 Prefer not to answer

38. What is your gender identity?

- ☐1 Male
- ☐2 Female
- ☐3 Non-binary
- ☐4 Other (please specify) ____________________________
- ☐5 Prefer not to answer

39. What is your race? (Check all that apply)

- ☐1 White
- ☐2 Black or African American
- ☐3 Asian
- ☐4 Native Hawaiian or Pacific Islander
- ☐5 American Indian or Alaskan Native
- ☐6 Other (please specify) ___________________

40. Are you Hispanic or Latino?

- ☐1 Yes
- ☐2 No

41. What is your current marital status?

- ☐1 Single, never married
- ☐2 Married or living as if married
- ☐3 Separated
- ☐4 Widowed
- ☐5 Divorced

42. Do you have children under the age of 18 years?

- ☐1 Yes
- ☐2 No

Thank you for completing The Internist Wellness Survey!
